# Genome Sequence of *Cronobacter sakazakii *SP291, a Persistent Thermotolerant Isolate Derived from a Factory Producing Powdered Infant Formula

**DOI:** 10.1128/genomeA.00082-13

**Published:** 2013-03-21

**Authors:** Karen A. Power, Qiongqiong Yan, Edward M. Fox, Shane Cooney, Séamus Fanning

**Affiliations:** UCD Centre for Food Safety, WHO-Collaborating Centre on Cronobacter, School of Public Health, Physiotherapy and Population Science, Molecular Innovation & Drug Discovery, University College Dublin, Belfield, Dublin, Ireland

## Abstract

*Cronobacter* is an opportunistic pathogen associated with meningitis in neonates. Based on long-term surveillance of a powdered infant formula production facility, a persistent and thermotolerant isolate, denoted *Cronobacter sakazakii* SP291, was detected. Here we report the complete genome along with the sequences of three plasmids identified in this organism.

## GENOME ANNOUNCEMENT

*Cronobacter* is a genus of Gram-negative, facultatively anaerobic, oxidase-negative, catalase-positive, rod-shaped bacteria of the family *Enterobacteriaceae*. This bacterium has been epidemiologically linked with cases of neonatal illness for which fatality rates ranging between 40 and 80% have been reported. In those cases in which the infected neonates recover, long-term sequelae include delayed neurological development, hydrocephalus, and permanent neurological damage.

Currently, seven species of *Cronobacter* are recognized: *Cronobacter sakazakii*, *C. malonaticus*, *C. turicensis*, *C. muytjensii*, *C. dublinensis*, *C. condimenti*, and *C. universalis* (formerly *C.* genomospecies 1). Currently, three completely closed sequenced genomes are available, two for *C. sakazakii* strain ATCC BAA-894 (NC_009778) (1) and strain ES15 (NC_017933) and one for *C. turicensis* strain LMG 23827 (NC_013282) (2). Molecular subtyping in a powdered infant formula (PIF) production site identified a persistent strain, denoted *C. sakazakii* SP291 (S. Cooney et al., submitted for publication). When this strain was studied in detail, the isolates enabled us to elaborate an interesting thermotolerant phenotype. To improve our understanding of this unique feature, we sequenced *C. sakazakii* SP291, and these data revealed a 4.3-Mb chromosome (57% GC) along with three resident plasmids of 118 (57% GC), 52 (49% GC), and 4.4 (54% GC) kb.

The sequence of the bacterial chromosome was generated using a combined approach, including both 8-kb paired-end Roche 454 FLX titanium pyrosequencing (carried out at the Centre for Genomic Research, University of Liverpool, Liverpool, United Kingdom) and 36-bp, 3-kb paired-end sequencing using the Illumina Genome Analyzer II platform (GATC Biotech, Konstanz, Germany). *De novo* sequence assembly of Illumina data was completed using Velvet v1.0.15 and VelvetOptimiser v2.1.7. Resulting contigs were split into 400-bp regions in order to combine these data from both platforms and make a final assembly using Newbler version 2.6 (Roche).

Plasmid sequencing was carried out using a Roche 454 genome sequencer (GS) FLX titanium series (Eurofins MWG, Ebersberg, Germany) and also assembled with Newbler v2.6.

Gap closure was accomplished using molecular cloning and traditional Sanger-based sequencing (Source Biosciences, Nottingham, United Kingdom).

All DNA sequences were subsequently annotated using the NCBI Prokaryotic Genomes Automatic Annotation Pipeline, and a detailed analysis will be included in a future publication.

### Nucleotide sequence accession numbers. 

The complete genome sequences of strain SP291 and the associated plasmids are available in GenBank under accession numbers CP004091-CP004094.
